# Effects of supplementation of *Bacillus amyloliquefaciens* on performance, systemic immunity, and intestinal microbiota of weaned pigs experimentally infected with a pathogenic enterotoxigenic *E. coli* F18

**DOI:** 10.3389/fmicb.2023.1101457

**Published:** 2023-03-15

**Authors:** Cynthia Jinno, Braden Wong, Martina Klünemann, John Htoo, Xunde Li, Yanhong Liu

**Affiliations:** ^1^Department of Animal Science, University of California, Davis, Davis, CA, United States; ^2^Evonik Operations GmbH, Hanau, Germany

**Keywords:** *Bacillus amyloliquefaciens*, *Escherichia coli* challenge, microbiome, performance, systemic immunity, weaned pigs

## Abstract

The objective of this study was to investigate the effects of dietary supplementation of *Bacillus (B.) amyloliquefaciens* on growth performance, diarrhea, systemic immunity, and intestinal microbiota of weaned pigs experimentally infected with F18 enterotoxigenic *Escherichia coli* (ETEC). A total of 50 weaned pigs (7.41 ± 1.35 kg BW) were individually housed and randomly allotted to one of the following five treatments: sham control (CON-), sham *B. amyloliquefaciens* (BAM-), challenged control (CON+), challenged *B. amyloliquefaciens* (BAM+), and challenged carbadox (AGP+). The experiment lasted 28 days, with 7 days of adaptation and 21 days after the first ETEC inoculation. ETEC challenge reduced (*P* < 0.05) average daily gain (ADG) of pigs. Compared with CON+, AGP+ enhanced (*P* < 0.05) ADG, while *B. amyloliquefaciens* supplementation tended (*P* < 0.10) to increase ADG in pigs from days 0 to 21 post-inoculation (PI). The ETEC challenge increased (*P* < 0.05) white blood cell (WBC) count on days 7 and 21 PI, while BAM+ pigs tended (*P* < 0.10) to have low WBC on day 7 PI and had lower (*P* < 0.05) WBC on day 21 PI compared with CON+. In comparison to AGP+ fecal microbiota, BAM+ had a lower (*P* < 0.05) relative abundance of *Lachnospiraceae* on day 0 and *Clostridiaceae* on day 21 PI, but a higher (*P* < 0.05) relative abundance of *Enterobacyeriaceae* on day 0. In ileal digesta, the Shannon index was higher (*P* < 0.05) in BAM+ than in AGP+. Bray-Curtis PCoA displayed a difference in bacterial community composition in ileal digesta collected from sham pigs vs. ETEC-infected pigs on day 21 PI. Pigs in BAM+ had a greater (*P* < 0.05) relative abundance of Firmicutes, but a lower (*P* < 0.05) relative abundance of Actinomycetota and Bacteroidota in ileal digesta than pigs in AGP+. Ileal digesta from AGP+ had a greater (*P* < 0.05) abundance of *Clostridium sensu stricto* 1 but lower (*P* < 0.05) *Bifidobacterium* than pigs in BAM+. In conclusion, supplementation of *B. amyloliquefaciens* tended to increase ADG and had limited effects on the diarrhea of ETEC-infected pigs. However, pigs fed with *B. amyloliquefaciens* exhibit milder systemic inflammation than controls. *B. amyloliquefaciens* differently modified the intestinal microbiota of weaned pigs compared with carbadox.

## Introduction

Newly weaned pigs experience a period of high stress from sudden environmental changes in housing and dietary changes from sow milk to a solid diet, which increases the risk of pigs experiencing post-weaning diarrhea induced by enterotoxigenic *Escherichia coli* (ETEC) (Fairbrother et al., 2005). Weaned pigs can experience watery diarrhea from ETEC disrupting the osmotic pressure in the intestines, leading pigs to undergo dehydration and reduced feed efficiency (Lee et al., 2016). Reduced feed intake equilibrates to reduced energy intake, which results in less growth and lower immunity in weaned pigs (Amezcua et al., 2002). Untreated pigs with post-weaning diarrhea can eventually lead to death, and the increasing mortality rate can negatively impact the economy of the swine industry. Therefore, ETEC pathogenicity must be suppressed within early contact.

In the swine industry, in-feed antibiotics were administered to treat post-weaning diarrhea and to prevent the spread of ETEC in weaned pigs. Some antibiotics, including carbadox, can be administered in-feed at a low dose to additionally promote pig growth and are commonly referred to as antibiotic growth promoters (AGP) (Lekagul et al., 2019). The continuous use of AGP increases the risk of antibiotic resistance, which can be transmitted zoonotically from pigs to humans, leading to increasing concerns for public health (Aarestrup, 2005). Thus, regulations and legislation have been applied in countries such as the European Union to ban or reduce the use of antibiotics in animal production, and the World Health Organization has developed a global action plan to increase awareness of using antibiotics for human health and livestock purposes (Casewell et al., 2003; World Health Organization, 2015). Although approximately 30 countries have restricted or banned the use of AGP, many other countries are still administering AGP in swine diets to prevent diarrhea and promote growth (Liao and Nyachoti, 2017). Hence, the swine industry is currently challenged to maintain health while improving the growth of newly weaned pigs without the use of AGP.

Categorized as direct-fed microbials, *Bacillus* spp. have shown to secrete secondary metabolites that may contribute to antimicrobial factors and can be easily isolated from soil (Sansinenea and Ortiz, 2011). In our previous study, *B. subtilis* DSM 25841 supplementation has been shown to reduce diarrhea and improve the growth performance of weaned pigs that were experimentally infected with ETEC (He et al., 2020b). *Bacillus* spp. also modify the intestinal microbiota when supplemented to pigs (Fouhse et al., 2016; Lee et al., 2019). Comparing the whole genomes, *B. subtilis* and *Bacillus amyloliquefaciens* have similar antibacterial synthetases, which lead *B. amyloliquefaciens* to also be categorized as direct-fed microbials (Koumoutsi et al., 2004). Salazar et al. (2017) have also identified bacteriocin-like substances in *B. amyloliquefaciens* that could inhibit the growth of pathogenic bacteria, and it has shown high-temperature resistance and pH stability, which may imply high survivability in the swine gastrointestinal tract. Findings in these previous studies suggest *B. amyloliquefaciens* as direct-fed microbials may have the potential to alleviate post-weaning diarrhea and enhance the growth performance of weaned pigs. However, limited research was reported on utilizing dietary *B. amyloliquefaciens* as in-feed supplementation on pig performance and intestinal health. Therefore, the present study aimed to investigate the effects of supplementing *B. amyloliquefaciens* on growth performance and systemic immunity of newly weaned pigs with or without the ETEC challenge and to compare the effects of *B. amyloliquefaciens* with carbadox on the fecal and ileal microbiota of weaned pigs.

## Materials and methods

### Animals and study design

The protocol for this experiment was reviewed and approved by the Institutional Animal Care and Use Committee (IACUC# 20809) at the University of California, Davis (UC Davis). A total of 50 weaned pigs [21 days old, 7.41 ±1.35 kg body weight (BW)] with an equal number of barrows and gilts were obtained from the Swine Teaching and Research Center, and the experiment was conducted at the Cole facility at UC Davis. All pigs and their sows did not receive *E. coli* vaccines, antibiotic injections, or antibiotics in feed prior to the experiment. After weaning, pigs were individually housed (pen size: 0.61 × 1.22 m) and assigned into one of 5 treatment groups with 10 replicate pigs per treatment using a randomized complete block design with sex normalized by BW and litter as blocks and pig as the experimental unit. There were four treatments in a 2 × 2 factorial arrangement with two diets [control (CON) vs. 0.10% inclusion rate with 10^9^ CFU/kg *B. amyloliquefaciens* (BAM) in the complete feed] and two challenges [sham (-) vs. ETEC (+)]. The fifth was an antibiotic (50 mg/kg of carbadox in the complete feed) treatment with an ETEC challenge (AGP+). Prior to weaning, tail samples were collected from all piglets to assess their susceptibility to ETEC F18 using the genotyping analysis described by Kreuzer et al. (2013). All pigs used in the present study were susceptible to ETEC F18.

The experiment included a 7-day habituation period and 21 days after the first ETEC F18 inoculation (day 0). A 2-phase feeding program was used, with weeks 1 and 2 as Phase I and weeks 3 and 4 as Phase II. Hence, six diets were prepared. Spray-dried plasma, antibiotics, and high levels of zinc oxide were not included in the diets. All diets were formulated to meet pig nutritional requirements (NRC, 2012; Table 1).

**Table 1:** Ingredient compositions of experimental diets, as fed basis^a^.

**Ingredient, %**	**Control, phase I**	**Control, phase II**
Corn	42.50	48.48
Dried whey	15.00	10.00
Soybean meal	20.00	24.00
Fish meal	4.00	3.00
Barley	10.00	10.00
Soy protein concentrate	3.00	-
Soybean oil	2.10	1.30
Limestone	0.95	0.95
DCP	0.55	0.52
L-Lysine·HCl	0.49	0.46
DL-Methionine	0.26	0.21
L-Threonine	0.22	0.20
L-Tryptophan	0.09	0.08
L-Valine	0.14	0.10
Salt	0.40	0.40
Vit-mineral, Sow 6^b^	0.30	0.30
Total	100.00	100.00
**Calculated energy and nutrient**		
Metabolizable energy, kcal/kg	3,364	3,310
Net energy, kcal/kg	2,526	2,480
Crude protein, %	20.54	19.77
Arg,^c^ %	1.14	1.11
His,^c^ %	0.47	0.46
Ile,^c^ %	0.76	0.72
Leu,^c^ %	1.50	1.44
Lys,^c^ %	1.42	1.32
Met,^c^ %	0.56	0.50
Thr,^c^ %	0.89	0.83
Trp,^c^ %	0.31	0.29
Val,^c^ %	0.97	0.89
Met + Cys,^c^ %	0.85	0.79
Phe + Tyr,^c^ %	1.36	1.32
Ca, %	0.83	0.75
Total *P*, %	0.66	0.60
Digestible *P*, %	0.43	0.36

a In each phase, two additional diets were formulated by adding 10^9^ CFU/kg B. amyloliquefaciens or 50 mg/kg of carbadox to the control diet, respectively.

b Provided by United Animal Health (Sheridan, IN). The premix provided the following quantities of vitamins and microminerals per kilogram of a complete diet: vitamin A as retinyl acetate, 11,136 IU; vitamin D3 as cholecalciferol, 2,208 IU; vitamin E as DL-alpha tocopheryl acetate, 66 IU; vitamin K as menadione dimethylprimidinol bisulfite, 1.42 mg; thiamin as thiamine mononitrate, 0.24 mg; riboflavin, 6.59 mg; pyridoxine as pyridoxine hydrochloride, 0.24 mg; vitamin B12, 0.03 mg; D-pantothenic acid as D-calcium pantothenate, 23.5 mg; niacin, 44.1 mg; folic acid, 1.59 mg; biotin, 0.44 mg; Cu, 20 mg as copper sulfate and copper chloride; Fe, 126 mg as ferrous sulfate; I, 1.26 mg as ethylenediamine dihydriodide; Mn, 60.2 mg as manganese sulfate; Se, 0.3 mg as sodium selenite and selenium yeast; and Zn, 125.1 mg as zinc sulfate.

c Amino acids are indicated as standardized ileal digestible AA.

Pigs in the ETEC challenge groups received 3 oral doses of ETEC F18 at 10^10^ CFU per dose. The F18 ETEC was cultured in Dr. Xunde Li's lab at the Western Institute for Food Safety and Security at UC Davis. The bacterial strain was originally isolated from a field disease outbreak by the University of Illinois Veterinary Diagnostic Lab (isolate number: U.IL-VDL # 05-27242), and the strain expresses heat-labile toxin, heat-stable toxin b, and Shiga-like toxins. On the terminating day of the experiment, all pigs were anesthetized by intramuscularly injecting 1 ml of a mixture of telazol (100 mg), ketamine (50 mg), and xylazine (50 mg) prior to an intracardiac injection of 78 mg sodium pentobarbital (Vortech Pharmaceuticals, Ltd., Dearborn, MI) per 1 kg of BW for euthanasia.

### Clinical observations and sample collection

Fecal and alertness scores were recorded two times daily from days 0 to 21 post-inoculation (PI). The fecal score was measured by two independent evaluators, with scores ranging from 1 to 5 (1, normal feces; 2, moist feces; 3, mild diarrhea; 4, severe diarrhea; and 5, watery diarrhea). The alertness score of each pig was also assessed visually, with the score ranging from 1 to 3 (1, normal; 2, slightly depressed or listless; and 3, severely depressed or recumbent). The frequency of diarrhea was calculated by quantifying the number of pigs and days with a fecal score ≥ 3 or ≥ 4, respectively.

Fecal samples were collected from the rectum of each pig at the beginning of the experiment (day −7), on day 0 before ETEC inoculation, and on days 2, 7, 14, and 21 PI to perform a fecal culture. Whole blood samples were collected from the jugular vein of all pigs on days −7 and 0, and days 7, 14, and 21 PI. Fresh blood samples were submitted to the Comparative Pathology Laboratory at the University of California, Davis, to measure total and differential blood cell counts. A multiparameter, automated, and programmed hematology analyzer (Drew/ERBA Scientific 950 FS Hematological Analyzer, Drew Scientific Inc., Miami, FL) was used for the assay to optimally differentiate porcine blood. Feeder weights, feed allowances, and pig BWs were recorded weekly to calculate average daily gain (ADG), average daily feed intake (ADFI), and gain-to-feed ratio (G:F) from days −7 to 0, days 0 to 7 PI, days 7 to 14 PI, and days 14 to 21 PI. Additional batches of fecal samples collected from days −7 and 0 before ETEC inoculation and on days 7, 14, and 21 PI and ileal digesta collected on day 21 PI were immediately frozen in liquid nitrogen and stored at −80°C until microbiota analysis.

### Fecal culture

Enterotoxigenic *E. coli* used in this experiment elaborates β-hemolysis and lactose fermentation, thus Columbia blood agar with 5% sheep blood and MacConkey agar were used to identify the percentage of β-hemolytic coliforms in feces. Fecal samples collected from the rectum of all pigs using cotton swabs on days 2, 7, 14, and 21 PI were used to perform fecal cultures. Briefly, fecal swabs were plated on Columbia blood agar and MacConkey agar using the quadrant streak plate method, and all plates were cultured in an air incubator at 37°C for 24 h. Total coliforms from both agars and β-hemolytic coliforms from blood agar were assessed visually using a scoring system ranging from 0 to 8 (0 = no bacterial growth, 8 = very heavy bacterial growth). The percentage of β-hemolytic coliforms in feces was calculated as the score ratio of β-hemolytic coliforms to total coliforms (Liu et al., 2013; He et al., 2020b).

### Microbiota analysis

Bacterial DNA was extracted from fecal samples and ileal digesta using the Quick-DNA Fecal/Soil Microbe Kit (Zymo Research, Irvine, CA, USA) according to the manufacturer's instructions. DNA samples were amplified in duplicates by PCR in the V4 region of the 16S rRNA gene using primers 515F (5′-XXXXXXXX**GT**GTGCCAGCMGCCGCGGTAA-3′), which included an 8-bp barcode (X) unique to each sample followed by a 2 nt Illumina adapter (bold), and 806R (5′-GGACTACHVGGGTWTCTAAT-3′) (Caporaso et al., 2012). The PCR reaction for each PCR well was composed of 2 μl of template DNA, 9.5 μl of nuclease-free water, 12.5 μl of GoTaq 2 × Master Mix (Promega, Madison, WI, USA), 0.5 μl of V4 reverse primer (10 μM), and 0.5 μl of barcoded forward primer (10 μM). Amplification was carried out in a thermocycler with the following settings: 94°C for 3 min for initializing denaturation; followed by 35 cycles of 94°C for 45 s, 50°C for 1 min, and 72°C for 1.5 min; and 72°C for 10 min for final elongation. The amplicon size for each sample was verified using agarose gel electrophoresis, and amplified samples were then pooled together, with the amount of sample added being quantified subjectively based on the band brightness in the agarose gel. The pooled sample was then purified using the QIAquick PCR Purification Kit (Qiagen, Hilden, Germany) and submitted to the UC Davis Genome Center DNA Technologies Core for 250 bp paired-end sequencing on the Illumina MiSeq platform (Illumina, Inc., San Diego, CA, USA).

Raw fastq files were first demultiplexed, and 8-bp barcodes were removed using saber (https://github.com/najoshi/sabre). Demultiplexed sequences were then imported into Quantitative Insights Into Microbial Ecology 2 (QIIME2; version 2020.8) to use the DADA2 plugin, which removes primers and lower-quality reads (Callahan et al., 2016; Bolyen et al., 2019, 2). Paired-end reads were denoised and merged, and chimeras were then removed to construct amplicon sequence variants (ASVs). Representative sequences for each ASV were aligned using MAFFT, and masked alignments were used to generate phylogenetic trees using FastTree2 (Price et al., 2010; Katoh and Standley, 2013). Python library scikit-learn was used to assign taxonomies based on representative sequences against Silva (version 138), which was pretrained in QIIME2 to be clipped in only the V4 hypervariable region and clustered at 99% sequence identity (Pedregosa et al., 2011; Quast et al., 2012; Bokulich et al., 2018).

### Statistical analyses

All data excluding microbiota were analyzed using SAS (SAS Inst. Inc., Cary, NC). The normality of all data was verified using the UNIVARIATE procedure. Values that deviated from the treatment mean by more than 3 times the interquartile range were assumed to be outliers and removed. Measurements were analyzed by ANOVA using the PROC MIXED of SAS in a randomized complete block design with pigs as experimental units. The model included treatment as the main effect and blocks as random effects. The LSMEANS statement and the PDIFF option of PROC MIXED were used to separate treatment means. Chi-square was used to find significance in the frequency of diarrhea. Statistical significance and tendency were assessed as α = 0.05 and α = 0.10, respectively.

Shannon and Chao1 indices were measured for alpha diversity by using the estimate_richness function in phyloseq (McMurdie and Holmes, 2013). Bray-Curtis matrix was used to compare community composition among treatments and day for feces and only treatment for ileal digesta. The relative abundance of each taxon in each sample was calculated by dividing the number of taxa by the total number of filtered reads in each sample. Files were exported from QIIME2 and imported into R 4.1.0 for data visualization and statistical analysis (R Core Team, 2021). All microbiota analyses were performed using the phyloseq package, and data were visualized using the ggplot2 package (Wickham, 2011). The normality and homoscedasticity were tested using the Shapiro-Wilks test and the Bartlett test, respectively. For fecal microbiota, the linear mixed-effect model was fitted using the lme4 package, with treatment, site, day, and interaction as fixed effects and pigs as random effects (Bates et al., 2014, p. 4). The significance of each term in the model was determined using the F-test as a type 3 analysis of variance using the ANOVA function in the car package, followed by a group comparison using the cld function in the emmeans package (Fox and Weisberg, 2018; Lenth, 2021). When normality or homoscedasticity was not observed, a nonparametric test was performed using the Kruskal-Wallis sum-rank test using the agricolae package (de Mendiburu and de Mendiburu, 2019). Bray-Curtis dissimilarity was first tested for homoscedasticity using the betadisper function and confirmed with *P* > 0.05. The statistical significance for beta diversity was then tested using PERMANOVA and the vegan package (Oksanen et al., 2013). Statistical significance was assessed as α = 0.05 and statistical tendency as α = 0.10. The *P-*values were adjusted for multiple comparisons using the false discovery rate (FDR).

## Results

### Growth performance, diarrhea, and white blood cell profile

No difference was observed in pig BW among treatments on days −7 and 0, day 7 PI, and day 14 PI (Table 2). On day 21 PI, pigs in BAM- had the heaviest BW, and pigs in CON+ had the lowest BW among all treatments (*P* < 0.05). Pig's final BW was greater (*P* < 0.05) in CON- than CON+, and final BW was greater (*P* < 0.05) in AGP+ than in CON+. No difference in ADG, ADFI, and gain:feed was observed in pigs between CON- and BAM- throughout the experiment. ETEC inoculation reduced (*P* < 0.05) ADG from days 0 to 21 PI and gain:feed from days 14 to 21 PI when CON+ was compared with CON-. Supplementation of AGP enhanced (*P* < 0.05) ADG from days 0 to 21 PI compared with CON+. Compared with CON+, pigs fed the BAM+ diet tended (*P* < 0.10) to increase ADFI and ADG of weaned pigs from days 0 to 21 PI and final BW at day 21 PI.

**Table 2:** Growth performance of weaned pigs fed a control (CON) diet or diets supplemented with *Bacillus amyloliquefaciens* (BAM) or antibiotics (AGP).

	**Sham**		***Escherichia coli***				
**Item** ^A^	**CON-**	**BAM-**	**CON**+	**BAM**+	**AGP**+	**SEM**	***P*** **-value**
**BW, kg**							
d −7	7.44	7.44	7.39	7.40	7.42	0.44	0.99
d 0	8.34	8.31	8.32	8.16	8.46	0.45	0.81
d 7 PI	9.75	9.60	9.15	8.95	9.62	0.47	0.18
d 14 PI	13.15	13.74	12.69	12.96	13.46	0.66	0.45
d 21 PI	17.92^ab^	18.09^a^	16.31^c^	16.63^bc^	17.65^ab^	0.67	<0.05
**ADG, g**							
d −7 to 0	128	140	130	108	149	16.22	0.53
d 0 to 7 PI	202^a^	184^a^	117^c^	132^bc^	163^ab^	16.59	<0.05
d 7 to 14 PI	486	594	506	570	552	36.36	0.094
d 14 to 21 PI	681^a^	622^a^	515^c^	525^bc^	603^ab^	30.97	<0.01
d 0 to 14 PI	344	390	312	365	360	22.12	0.102
d 0 to 21 PI	456^a^	467^a^	379^c^	403^bc^	441^ab^	22.14	<0.05
**ADFI, g**							
d −7 to 0	326	264	266	295	347	25.99	0.104
d 0 to 7 PI	716^ab^	829^a^	616^bc^	679^bc^	588^c^	49.36	<0.01
d 7 to 14 PI	843	867	751	897	831	50.42	0.31
d 14 to 21 PI	1,066	1,084	856	1,052	1,091	67.17	0.38
d 0 to 14 PI	779^ab^	881^a^	702^b^	786^ab^	746^b^	37.95	<0.05
d 0 to 21 PI	875^ab^	927^a^	788^b^	879^ab^	816^b^	30.90	<0.05
**Gain:Feed**							
d −7 to 0	0.38	0.40	0.49	0.37	0.42	0.047	0.46
d 0 to 7 PI	0.29	0.23	0.20	0.18	0.24	0.040	0.37
d 7 to 14 PI	0.60	0.69	0.68	0.69	0.67	0.035	0.35
d 14 to 21 PI	0.62^a^	0.63^a^	0.53^b^	0.51^b^	0.56^ab^	0.025	<0.01
d 0 to 14 PI	0.45	0.46	0.45	0.46	0.50	0.036	0.75
d 0 to 21 PI	0.53	0.51	0.48	0.48	0.52	0.021	0.29

A BW, body weight; ADG, average daily gain; ADFI, average daily feed intake; PI, post-inoculation.

Each least squares mean represents 9–10 observations.

^a − c^Means without a common superscript are different (*P* < 0.05).

**Figure 1:**
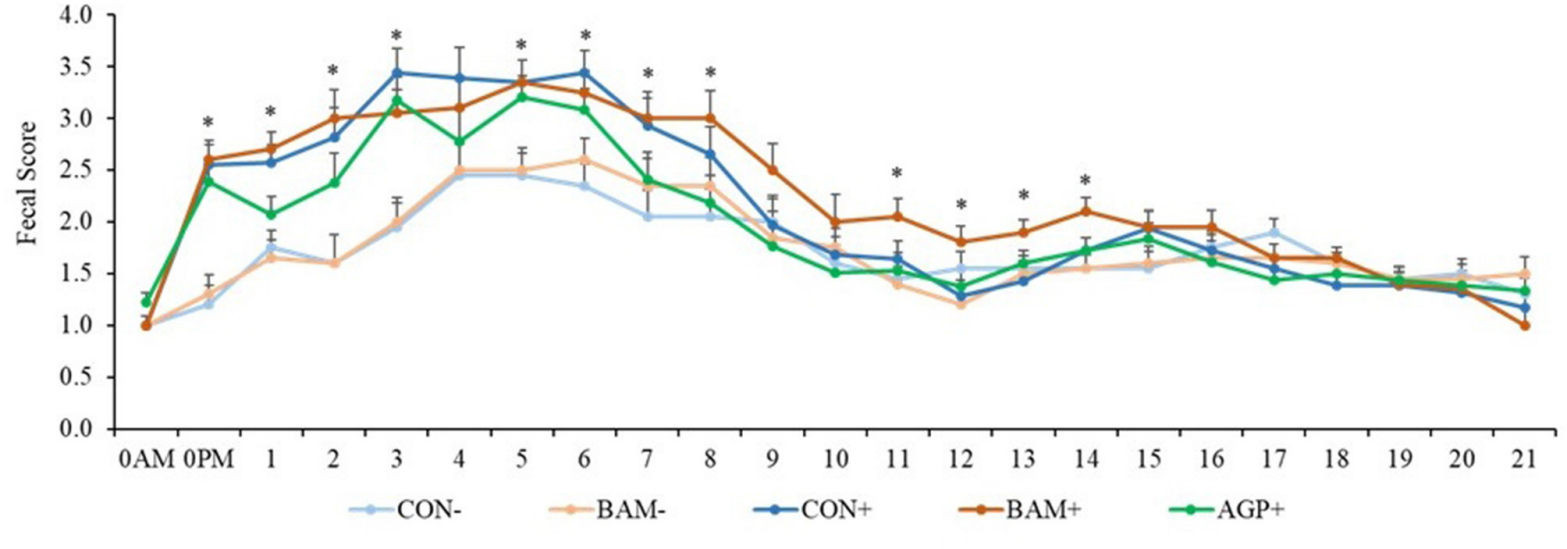
The daily fecal score of weaned pigs fed diets supplemented with *Bacillus amyloliquefaciens* (BAM) or antibiotics (AGP) with or without enterotoxigenic *Escherichia coli* challenge. Fecal score = 1, normal feces; 2, moist feces; 3, mild diarrhea; 4, severe diarrhea; 5, watery diarrhea. PI, post-inoculation. ^*^P < 0.05, indicating diarrhea scores were different among treatments. Each least squares mean represents 9–10 observations.

**Figure 2:**
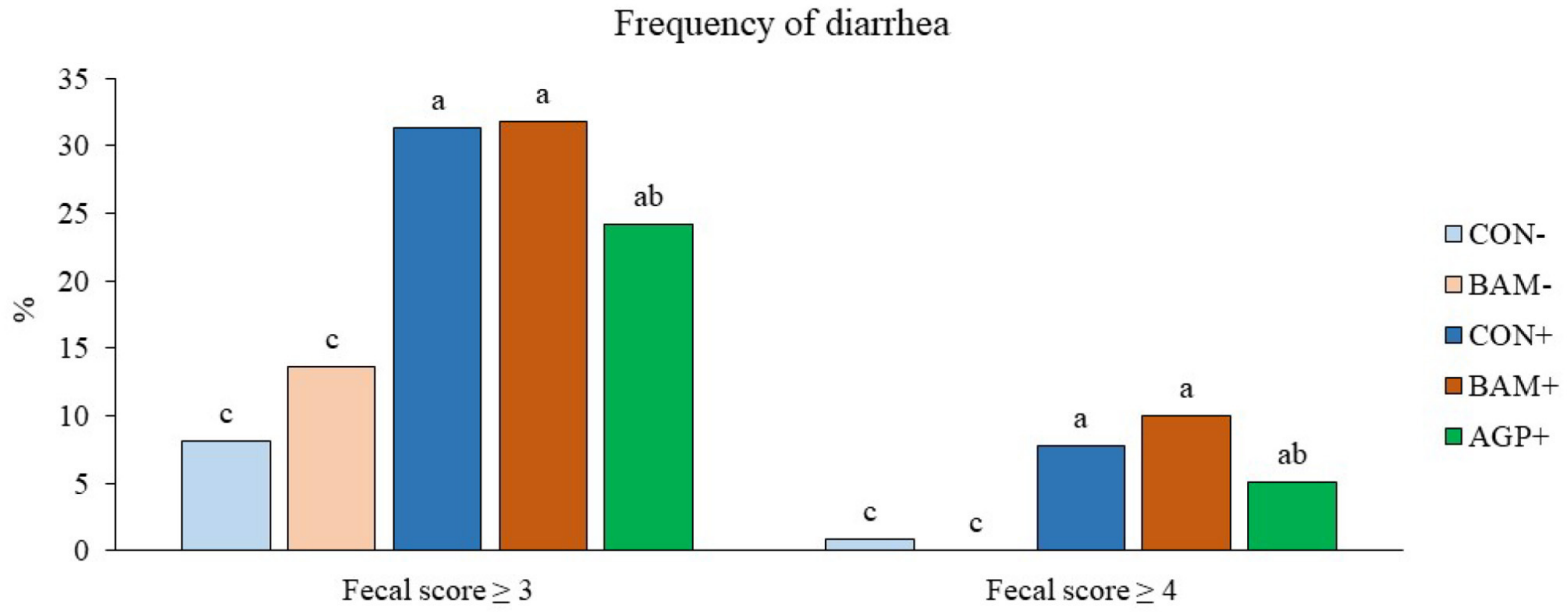
Frequency of diarrhea in weaned pigs fed diets supplemented with *Bacillus amyloliquefaciens* (BAM) or antibiotics (AGP). The frequency of diarrhea was calculated as the percentage of pig days with a fecal score ≥ 3 or ≥ 4 in the total number of pig days. Each least squares mean represents 9–10 observations. ^a−*c*^Means without a common superscript are different (*P* < 0.05).

Pigs in the sham groups (CON- and BAM-) had the lowest fecal score throughout the experiment (Figure 1). Pigs in BAM+ and CON+ had a greater (*P* < 0.05) fecal score from days 1 to 8 PI than pigs in CON-, while pigs in AGP+ were intermediate. While for all treatments the fecal score decreased as of day 9 to a level below diarrhea, between days 11 and 14 PI, pigs in BAM+ had the highest (*P* < 0.05) fecal score among treatments. After ETEC inoculation, the frequency of diarrhea (diarrhea score ≥ 3) was 31.36% in CON+ pigs, while the diarrhea frequency was 8.18% in CON- pigs (Figure 2). Pigs in CON+, BAM+, and AGP+ had higher (*P* < 0.05) frequency of diarrhea than pigs in CON- and BAM-, regardless of incidence (diarrhea score ≥ 3) and severity (diarrhea score ≥ 4). Supplementation of either BAM or AGP did not affect the frequency of diarrhea throughout the experiment, although pigs in AGP+ had a numerically lower frequency of diarrhea than pigs in CON+ and BAM+.

**Figure 3:**
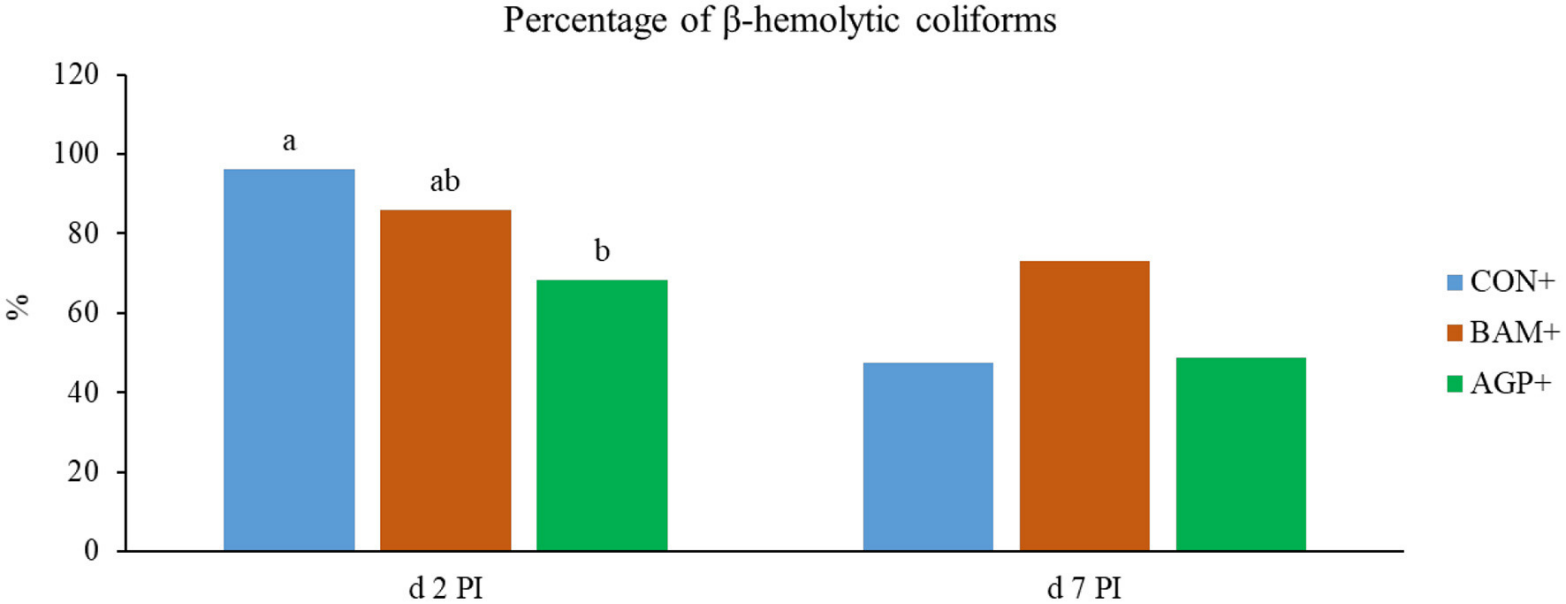
The percentage (%) of β-hemolytic total coliforms in fecal samples of *Escherichia coli* challenged weaned pigs fed a control diet (CON+) or diets supplemented with *Bacillus amyloliquefaciens* (BAM+) or antibiotics (AGP+). No β-hemolytic coliforms were observed in the fecal samples of pigs in the sham groups. No β-hemolytic coliforms were observed in the fecal samples of pigs before *E. coli* (ETEC) challenge and on days 14 and 21 post-inoculation (PI). Each least squares mean represents 9–10 observations. ^a, b^Means without a common superscript are different (*P* < 0.05).

No β-hemolytic coliforms were detected in fecal samples of pigs in CON- and BAM- throughout the experiment, and no β-hemolytic coliforms were observed in all pigs on days −7 and 0, day 14 PI, and day 21 PI. On day 2 PI, AGP+ had a lower (*P* < 0.05) percentage of β-hemolytic coliforms proliferated on the blood agar plates than CON+ (Figure 3). On day 7 PI, no difference was observed among all three treatments under the ETEC challenge.

No difference was observed in the white blood cell profile among the five treatments on day −7 (Table 3). On day 0, lymphocyte count was greater (*P* < 0.05) in AGP+ than in other treatments, except for BAM+. However, lymphocyte percentage was greater (*P* < 0.05) in BAM+ than in CON- and CON+. After ETEC inoculation, greater (*P* < 0.05) white blood cell and lymphocyte counts were observed in CON+ on days 7 and 21 PI, and a greater (*P* < 0.05) neutrophil count was observed in CON+ on day 14 PI, compared with CON-. Pigs in BAM+ had lower (*P* < 0.05) neutrophils on day 14 PI and lower (*P* < 0.05) white blood cells, neutrophils, and lymphocytes on day 21 PI, than pigs in CON+. Supplementation of AGP reduced (*P* < 0.05) neutrophil count on days 14 and 21 PI and increased (*P* < 0.05) lymphocyte percentage and monocyte percentage on day 21 PI compared with CON+. No difference was observed in the white blood cell profile of pigs between BAM+ and AGP+, with the exception that pigs in AGP+ had a greater (*P* < 0.05) monocyte percentage on days 7 and 21 PI but lower (*P* < 0.05) neutrophil percentage on day 21 PI than pigs in BAM+. No difference was observed in the white blood cell profile of pigs between BAM- and CON-. No difference was observed in the red blood cell profile of pigs among all treatments (Data were not shown).

**Table 3:** Total and differential white blood cells in weaned pigs a fed control (CON) diet or diets supplemented with *Bacillus amyloliquefaciens* (BAM) or antibiotics (AGP).

	**Sham**		***Escherichia coli***				
**Item** ^A^	**CON-**	**BAM-**	**CON**+	**BAM**+	**AGP**+	**SEM**	***P*** **-value**
**d** −**7**							
WBC, 10^3^/μL	9.86	7.94	8.66	8.91	9.88	1.55	0.82
Neu, 10^3^/μL	3.51	2.91	3.83	3.57	4.14	0.58	0.52
Lym, 10^3^/μL	6.10	4.52	4.22	4.77	5.06	0.85	0.58
Mono, 10^3^/μL	0.57	0.45	0.57	0.53	0.53	0.12	0.96
Eos, 10^3^/μL	0.027	0.041	0.048	0.033	0.092	0.023	0.35
Baso, 10^3^/μL	0.017	0.009	0.016	0.010	0.032	0.011	0.57
Neu, %	33.51	37.38	44.90	40.08	41.61	2.39	0.051
Lym, %	60.62	56.02	47.88	53.26	51.48	2.78	0.067
Mono, %	5.58	5.93	6.39	6.02	5.79	1.01	0.98
Eos, %	0.19	0.52	0.61	0.45	0.85	0.24	0.47
Baso, %	0.116	0.093	0.235	0.126	0.292	0.098	0.58
Neu:Lym	0.57	0.72	0.95	0.79	0.88	0.095	0.12
**d 0**							
WBC, 10^3^/μL	10.41	9.17	11.25	11.21	13.20	1.14	0.13
Neu, 10^3^/μL	4.86	3.82	5.45	4.26	5.36	0.62	0.21
Lym, 10^3^/μL	4.86^b^	4.75^b^	5.23^b^	6.31^ab^	7.14^a^	0.64	<0.05
Mono, 10^3^/μL	0.60	0.56	0.46	0.56	0.55	0.095	0.87
Eos, 10^3^/μL	0.067	0.045	0.078	0.061	0.095	0.020	0.50
Baso, 10^3^/μL	0.021	0.008	0.035	0.022	0.046	0.011	0.16
Neu, %	46.59	42.18	47.80	37.21	40.37	3.10	0.067
Lym, %	46.73^b^	51.36^ab^	46.96^b^	57.28^a^	54.45^ab^	3.08	<0.05
Mono, %	5.65	5.90	4.14	4.77	4.21	0.68	0.17
Eos, %	0.74	0.48	0.74	0.56	0.66	0.20	0.86
Baso, %	0.29	0.077	0.37	0.19	0.31	0.12	0.43
Neu:Lym	1.17	0.86	1.05	0.70	0.83	0.15	0.15
**d 7 PI**							
WBC, 10^3^/μL	11.45^b^	13.17^ab^	17.03^a^	15.92^ab^	16.14^a^	1.18	<0.05
Neu, 10^3^/μL	5.34	5.74	7.15	6.76	6.50	0.76	0.34
Lym, 10^3^/μL	5.28^b^	6.21^ab^	8.71^a^	8.26^a^	8.22^a^	0.94	<0.05
Mono, 10^3^/μL	0.81	1.00	0.93	0.67	1.03	0.146	0.38
Eos, 10^3^/μL	0.052	0.139	0.186	0.161	0.311	0.074	0.15
Baso, 10^3^/μL	0.021	0.022	0.050	0.057	0.062	0.016	0.17
Neu, %	46.06	44.16	42.44	42.15	40.77	2.40	0.55
Lym, %	46.21	47.06	50.65	52.71	50.59	2.50	0.33
Mono, %	7.11^a^	7.46^a^	5.66^ab^	3.94^b^	6.39^a^	0.76	<0.05
Eos, %	0.47	1.06	1.00	0.92	1.81	0.43	0.22
Baso, %	0.19	0.17	0.27	0.35	0.35	0.085	0.24
Neu:Lym	1.03	0.99	0.88	0.83	0.84	0.096	0.44
**d 14 PI**							
WBC, 10^3^/μL	16.29	16.86	19.19	17.26	18.25	1.51	0.49
Neu, 10^3^/μL	7.62^b^	7.22^b^	9.49^a^	7.92^b^	7.27^b^	0.51	<0.05
Lym, 10^3^/μL	7.79	8.36	8.75	8.21	9.48	1.18	0.65
Mono, 10^3^/μL	0.67	0.67	0.63	0.54	0.80	0.086	0.32
Eos, 10^3^/μL	0.24	0.49	0.28	0.55	0.62	0.15	0.28
Baso, 10^3^/μL	0.019	0.043	0.043	0.092	0.090	0.022	0.071
Neu, %	47.24	43.76	49.56	46.10	40.99	3.23	0.12
Lym, %	47.33	49.18	45.34	47.24	50.81	3.24	0.47
Mono, %	4.07	4.02	3.41	3.13	4.53	0.46	0.20
Eos, %	1.29	2.78	1.44	3.03	3.24	0.80	0.28
Baso, %	0.11	0.23	0.23	0.52	0.45	0.15	0.087
Neu:Lym	1.02	0.93	1.17	0.99	0.85	0.15	0.30
**d 21 PI**							
WBC, 10^3^/μL	11.86^b^	12.06^ab^	14.53^a^	10.25^b^	12.05^ab^	1.19	<0.05
Neu, 10^3^/μL	5.45^ab^	5.49^ab^	6.33^a^	4.13^bc^	4.07^c^	0.54	<0.01
Lym, 10^3^/μL	5.59^b^	5.63^b^	7.30^a^	5.36^b^	6.79^ab^	0.69	<0.05
Mono, 10^3^/μL	0.52	0.58	0.50	0.38	0.69	0.081	0.18
Eos, 10^3^/μL	0.26	0.33	0.32	0.29	0.43	0.086	0.70
Baso, 10^3^/μL	0.044	0.040	0.076	0.079	0.067	0.018	0.20
Neu, %	45.39^a^	45.11^a^	43.59^a^	41.07^a^	34.15^b^	1.96	<0.01
Lym, %	47.63^b^	46.91^b^	50.39^b^	51.83^ab^	56.28^a^	1.88	<0.01
Mono, %	4.40^abc^	4.88^ab^	3.28^c^	3.72^bc^	5.74^a^	0.57	<0.05
Eos, %	2.18	2.77	2.18	2.75	3.33	0.60	0.66
Baso, %	0.39^b^	0.34^b^	0.51^ab^	0.76^a^	0.51^ab^	0.10	<0.05
Neu:Lym	0.98^a^	0.99^a^	0.89^a^	0.82^ab^	0.62^b^	0.072	<0.01

A PI, post-inoculation; WBC, white blood cell; Neu, neutrophil; Lym, lymphocyte; Mono, monocyte; Eos, eosinophil; Baso, basophil.

Each least squares mean represents 9–12 observations.

^a−*c*^Means without a common superscript are different (*P* < 0.05).

### Fecal microbiota

**Figure 4:**
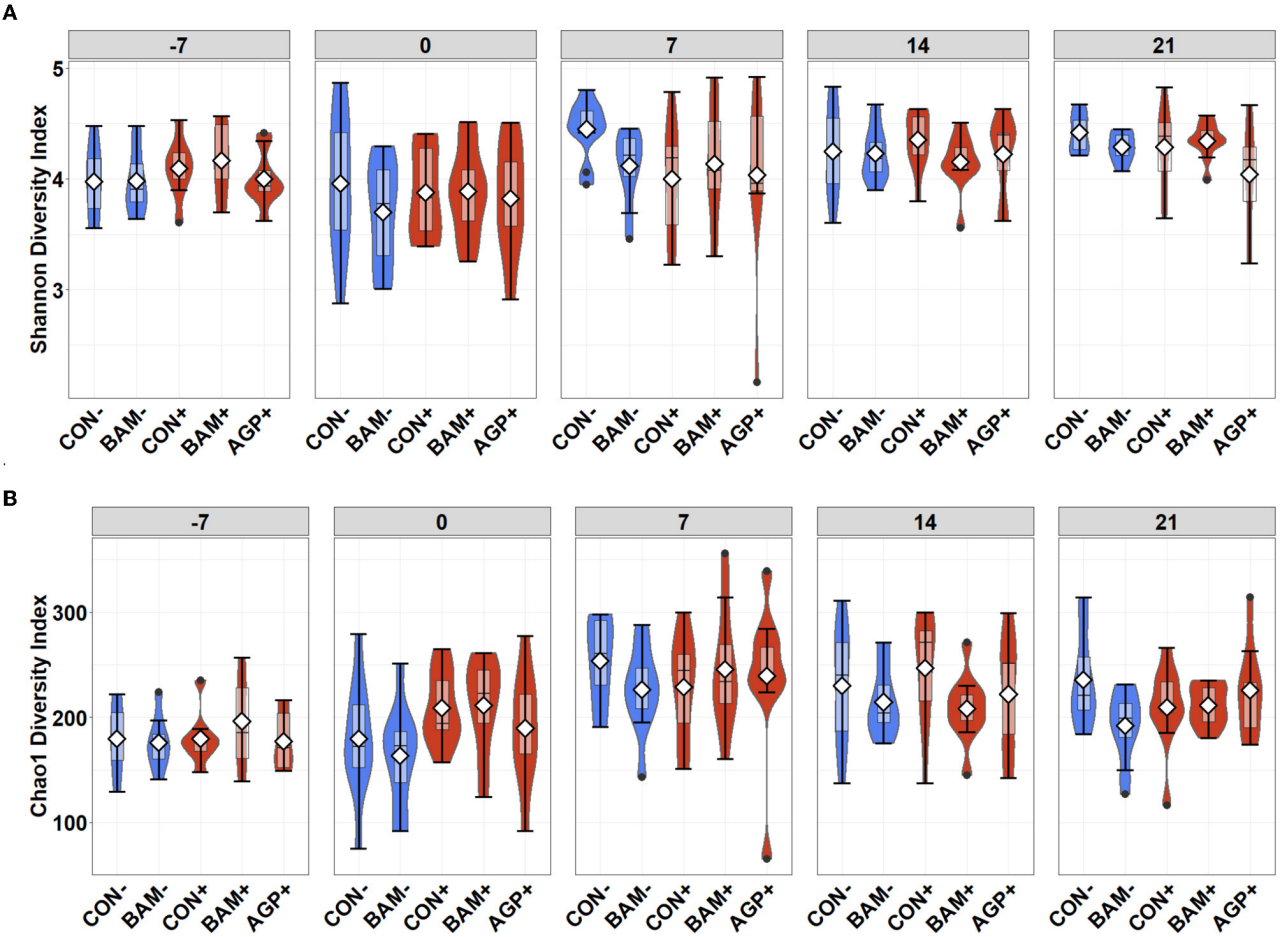
Alpha diversity as indicated by Shannon **(A)** and Chao1 **(B)** indices in feces of weaned pigs fed with control (CON) diet or diets supplemented with *Bacillus amyloliquefaciens* (BAM), or antibiotics (AGP) at the beginning of the experiment (day−7), the first day of *E. coli* (ETEC) inoculation (day 0), and days 7, 14, and 21 post-inoculation. No difference was observed in Shannon **(A)** and Chao1 **(B)** indices among treatments. Violin plots are colored whether not infected (blue) or infected with *E. coli* (red). Data are expressed as mean (diamond) ± SEM.

**Figure 5:**
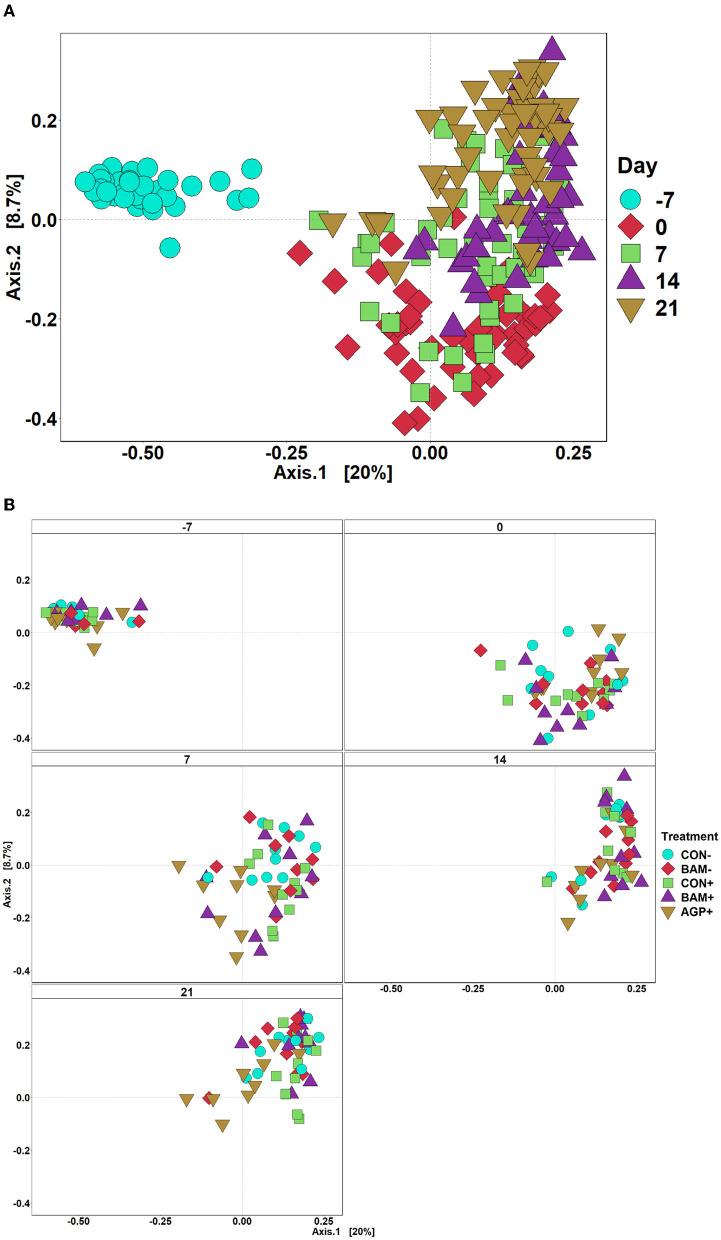
Principal coordinate analysis (PCoA) based on Bray-Curtis distance for beta diversity of fecal samples of weaned pigs fed with a control (CON) diet or diets supplemented with *Bacillus amyloliquefaciens* (BAM) or antibiotics (AGP). Different symbols and shapes represent day fecal samples collected on days−7 and 0 before *E. coli* (ETEC) inoculation and days 7, 14, and 21 post-inoculation **(A)**. Different symbols and shapes represent treatments **(B)**. Each treatment has 9–10 observations.

The mean number of reads was 15,368, and the total number of taxa identified was 4,410 in the sequence data. An increase (*P* < 0.05) in Shannon and Chao1 indices was observed in feces when the age of the pig increased from day −7 to day 21 PI. However, no significant differences in Shannon and Chao1 indices were observed in feces among treatments throughout the experiment (Figure 4). In beta diversity, fecal samples collected on day −7 were clustered and separated from fecal samples collected on days 0, 7, 14, and 21 PI (Figure 5A). Fecal samples collected on day 0 were clustered away from fecal samples collected on day 21 PI. Fecal samples from all treatments were clustered together on day −7 (Figure 5B). On day 7 PI, fecal samples from AGP+ were moderately clustered away from CON- and CON+. AGP+ was clustered farther away from BAM+ and CON+ on day 21 PI.

The three most abundant phyla (most to least abundant) were Firmicutes, Bacteroidota, and Proteobacteria in the fecal samples of pigs from all treatments throughout the experiment. No difference was observed in the relative abundance of phyla in fecal samples on day −7 (Table 4). The relative abundance of Firmicutes was the highest on day 14 PI compared with other time points. The relative abundance of Proteobacteria was observed to be the highest (*P* < 0.05) on day 7 PI and the relative abundance of Bacteroidota was the highest (*P* < 0.05) on day 21 PI among all fecal collection days. At the family level, the relative abundance of *Bacteroidaceae* and *Clostridiaceae* was decreased (*P* < 0.05), while the relative abundance of *Prevotellaceae, Lachnospiraceae*, and *Lactobacillaceae* was increased (*P* < 0.05) in feces as the age of pig increased.

**Table 4:** Relative abundance (%) of Bacteroidota, Firmicutes, and Proteobacteria and their top families in feces of weaned pigs a fed control (CON) diet or diets supplemented with *Bacillus amyloliquefaciens* (BAM) or antibiotics (AGP).

	**Sham**		***Escherichia coli***		
	**CON-**	**BAM-**	**CON**+	**BAM**+	**AGP**+
**d** −**7**					
Bacteroidota	16.78	13.05	14.35	14.68	12.09
*Bacteroidaceae*	6.11	3.59	6.40	5.35	3.64
*Muribaculaceae*	1.41	1.93	1.84	1.18	1.47
*Prevotellaceae*	3.96	2.29	2.31	2.75	2.36
*Rikenellaceae*	3.35	2.79	2.39	2.67	2.63
Firmicutes	58.19	65.1	63.87	61.37	68.43
*Clostridiaceae*	11.42	10.56	7.93	8.96	10.31
*Lachnospiraceae*	8.36	8.45	10.45	10.24	9.38
*Lactobacillaceae*	2.80	5.45	5.39	3.08	8.10
*Oscillospiraceae*	5.58	6.01	10.49	7.27	8.26
*Ruminococcaceae*	10.8	11.78	6.03	7.22	10.33
Proteobacteria	3.29	4.54	2.33	4.18	2.28
*Enterobacteriaceae*	1.31	3.26	1.79	3.04	1.66
*Succinivibionaceae*	1.83	1.03	0.38	1.01	0.48
**d 0**					
Bacteroidota	13.67	9.67	12.38	14.04	15.02
*Bacteroidaceae*	0.41^ab^	0.36^ab^	0.19^b^	0.46^a^	0.34^ab^
*Muribaculaceae*	3.13	1.92	2.60	3.09	2.35
*Prevotellaceae*	8.09	5.92	7.60	7.39	10.67
*Rikenellaceae*	1.33	1.12	1.52	2.11	1.30
Firmicutes	76.45	79.61	76.99	75.61	76.59
*Clostridiaceae*	1.16^a^	0.13^b^	0.51^a^	0.15^ab^	0.33^ab^
*Lachnospiraceae*	18.08^b^	18.91^b^	19.61^ab^	16.50^b^	27.29^a^
*Lactobacillaceae*	29.36	36.43	32.27	35.40	25.55
*Oscillospiraceae*	5.44	4.22	4.66	4.72	3.38
*Ruminococcaceae*	4.46	4.62	4.90	4.52	5.68
Proteobacteria	1.52	1.96	0.90	1.44	0.39
*Enterobacteriaceae*	1.21^a^	0.65^ab^	0.61^ab^	1.21^a^	0.20^b^
*Succinivibionaceae*	0.26	0.88	0.15	0.16	0.16
**d 7 PI**					
Bacteroidota	17.2^ab^	15.75^ab^	12.82^b^	15.27^ab^	18.61^a^
*Bacteroidaceae*	0.27^ab^	0.17^b^	0.32^ab^	0.64^ab^	1.34^a^
*Muribaculaceae*	4.80	3.64	3.16	2.66	4.72
*Prevotellaceae*	9.34	9.62	7.84	9.57	8.84
*Rikenellaceae*	1.60	1.56	1.10	1.39	1.40
Firmicutes	67.71	69.08	69.46	64.55	56.64
*Clostridiaceae*	0.21	0.11	0.16	0.45	0.69
*Lachnospiraceae*	29.76^a^	28.23^ab^	30.83^a^	25.14^ab^	16.48^b^
*Lactobacillaceae*	7.99^b^	11.59^ab^	17.39^a^	16.05^ab^	17.01^a^
*Oscillospiraceae*	6.40	3.16	4.19	4.57	6.72
*Ruminococcaceae*	5.89	8.17	4.17	5.5	3.39
Proteobacteria	5.42	7.24	9.86	9.72	16.54
*Enterobacteriaceae*	1.82^b^	5.68^ab^	8.84^ab^	7.49^ab^	15.9^a^
*Succinivibionaceae*	3.36	1.06	0.87	1.65	0.50
**d 14 PI**					
Bacteroidota	15.64	12.19	17.71	17.93	17.53
*Bacteroidaceae*	0.02^ab^	0.03^ab^	0.00^b^	0.34^a^	0.13^ab^
*Muribaculaceae*	3.3	2.61	2.56	1.16	3.20
*Prevotellaceae*	11.10^ab^	8.44^b^	14.08^a^	15.95^a^	12.47^ab^
*Rikenellaceae*	0.82	0.89	0.99	0.39	1.39
**Firmicutes**					
*Clostridiaceae*	77.87	78.45	72.09	71.78	73.29
*Lachnospiraceae*	0.74	0.45	0.57	0.31	3.24
*Lactobacillaceae*	26.08	27.29	23.64	24.70	25.49
*Oscillospiraceae*	10.89	15.79	11.75	15.42	15.26
*Ruminococcaceae*	3.63	2.51	4.03	1.59	5.74
Proteobacteria	1.24	0.95	2.55	1.35	1.14
*Enterobacteriaceae*	0.10	0.24	0.03	0.15	0.02
*Succinivibionaceae*	1.11	0.68	2.37	1.17	1.03
**d 21 PI**					
Bacteroidota	20.21	22.24	16.50	19.47	18.34
*Bacteroidaceae*	0.01	0.01	0.01	0.01	0.16
*Muribaculaceae*	2.98	3.03	4.19	3.19	5.28
*Prevotellaceae*	15.97^ab^	17.91^a^	10.78^b^	15.09^ab^	11.33^b^
*Rikenellaceae*	1.05	1.11	1.36	1.10	1.26
Firmicutes	67.12^ab^	61.62^b^	73.47^a^	68.29^ab^	66.89^ab^
*Clostridiaceae*	1.39^b^	1.08^b^	0.73^b^	4.12^b^	18.48^a^
*Lachnospiraceae*	20.98	18.42	23.19	18.5	17.16
*Lactobacillaceae*	9.12^ab^	7.33^ab^	12.38^a^	8.75^ab^	6.20^b^
*Oscillospiraceae*	3.63	3.00	4.28	4.04	7.38
*Ruminococcaceae*	6.91	7.69	7.00	7.74	5.36
Proteobacteria	3.27	3.36	1.61	2.7	6.41
*Enterobacteriaceae*	0.05	0.05	0.01	0.03	0.02
*Succinivibionaceae*	2.89	3.20	1.56	2.61	6.28

^a, b^Means without a common superscript are different (*P* < 0.05).

Each mean represents 9–10 observations.

Dietary treatments had limited effects on the relative abundance of Firmicutes, Bacteroidota, and Proteobacteria in fecal samples collected on day 0, except that pigs in AGP+ had the greatest (*P* < 0.05) *Lachnospiraceae* but the lowest (*P* < 0.05) *Enterobacteriaceae* among all treatments. Pigs in BAM- had a relatively lower (*P* < 0.05) abundance of *Clostridiaceae* than pigs in CON-, while pigs in BAM+ had a relatively higher (*P* < 0.05) abundance of *Bacteroidaceae* than pigs in CON- on day 0. ETEC inoculation increased (*P* < 0.05) the relative abundance of *Lactobacillaceae* in feces on day 7 PI when CON+ was compared with CON-. Supplementation of AGP enhanced (*P* < 0.05) the relative abundance of Bacteroidota on day 7 PI and *Clostridiaceae* on day 21 PI, but AGP decreased (*P* < 0.05) the relative abundance of *Lachnospiraceae* on day 7 PI and *Lactobacillaceae* on day 21 PI compared with CON+. Pigs in AGP+ also had a greater (*P* < 0.05) relative abundance of *Clostridiaceae* in feces than pigs in BAM+ on day 21 PI.

Within the 12 most abundant genera, nine genera were classified under Firmicutes, two under Bacteroidota, and one under Proteobacteria (Table 5). The three most abundant genera in all fecal samples throughout the experiment were *Lactobacillus, Blautia*, and *Prevotella*. The relative abundance of these three genera was increased from days −7 to 0. However, the relative abundance of *Lactobacillus* in fecal samples from all pigs decreased (*P* < 0.05) from days 0 to 7 PI. No difference was observed in the most abundant genera in fecal samples of pigs between CON- and BAM- throughout the experiment. On day 0, pigs supplemented with AGP had a greater (*P* < 0.05) relative abundance of *Prevotella* than pigs in BAM+ and BAM- and had a greater (*P* < 0.05) relative abundance of *Blautia* than pigs in all other treatments. Compared CON+ with CON-, ETEC infection enhanced (*P* < 0.05) the relative abundance of *Lactobacillus* on day 7 PI and *Megasphaera* on day 21 PI but reduced (*P* < 0.05) the relative abundance of *Coprococcus* on day 14 PI and *Streptococcus* on day 21 PI. Supplementation of AGP reduced (*P* < 0.05) the relative abundance of *Agathobacter* on day 7 PI, the relative abundance of *Dorea* and *Streptococcus* on day 14 PI, and the relative abundance of *Blautia, Dorea, Lactobacillus*, and *Megasphaera* on day 21 PI compared with CON+. Compared with AGP+, pigs in BAM+ had a relatively higher (*P* < 0.05) abundance of *Streptococcus* on day 14 PI, and *Prevotella, Megasphaera*, and *Streptococcus* on day 21 PI in feces.

**Table 5:** Relative abundance (%) of most abundant genera from Bacteroidota, Firmicutes, and Proteobacteria in feces of weaned pigs fed control (CON) diet or diets supplemented with *Bacillus amyloliquefaciens* (BAM) or antibiotics (AGP).

	**Sham**		***Escherichia coli***		
	**CON-**	**BAM-**	**CON**+	**BAM**+	**AGP**+
**d** −**7**					
**Bacteroidota**					
*Muribaculaceae*	1.41	1.93	1.84	1.18	1.47
*Prevotella*	0.76	0.33	0.28	0.86	0.71
**Firmicutes**					
*Agathobacter*	0.01	0.06	0.01	0.17	0.00
*Blautia*	0.07	0.16	0.09	0.11	0.16
*Coprococcus*	0.01	0.18	0.00	0.18	0.04
*Dorea*	0.00	0.01	0.00	0.00	0.00
*Faecalibacterium*	0.01	0.03	0.00	0.00	0.00
*Lactobacillus*	2.8	5.45	5.39	3.08	8.1
*Megasphaera*	0.33	0.63	0.08	0.91	0.24
*Ruminococcus*	9.76	10.07	4.52	5.92	7.82
*Streptococcus*	0.18	0.86	0.66	0.68	0.4
**Proteobacteria**					
*Escherichia-Shigella*	1.31	3.26	1.79	3.04	1.66
**d 0**					
**Bacteroidota**					
*Muribaculaceae*	3.13	1.92	2.6	3.09	2.35
*Prevotella*	4.52^ab^	2.65^b^	3.92^ab^	3.19^b^	6.74^a^
**Firmicutes**					
*Agathobacter*	0.91	1.75	1.74	3.31	3.56
*Blautia*	4.51^b^	4.41^b^	4.81^b^	2.73^b^	12.00^a^
*Coprococcus*	1.49	1.39	1.50	1.78	2.33
*Dorea*	1.09	0.47	0.65	0.34	0.34
*Faecalibacterium*	1.39	1.39	1.34	1.61	1.49
*Lactobacillus*	29.36	36.43	32.27	35.4	25.51
*Megasphaera*	3.04	5.59	3.65	3.96	5.66
*Ruminococcus*	0.84	0.69	0.63	0.81	0.94
*Streptococcus*	1.00^a^	0.16^ab^	0.25^b^	0.12^b^	0.18^ab^
**Proteobacteria**					
*Escherichia-Shigella*	1.21^a^	0.65^a^	0.61^a^	1.21^a^	0.2^b^
**d 7 PI**					
**Bacteroidota**					
*Muribaculaceae*	4.80	3.64	3.16	2.66	4.72
*Prevotella*	4.21	5.32	3.49	5.00	3.36
**Firmicutes**					
*Agathobacter*	2.59^a^	4.29^a^	2.06^a^	1.89^ab^	0.9^b^
*Blautia*	11.37^a^	9.19^a^	9.57^ab^	6.00^b^	3.17^b^
*Coprococcus*	2.10	1.95	3.04	3.40	1.78
*Dorea*	4.11^a^	3.19^a^	2.97^ab^	1.20^b^	1.08^b^
*Faecalibacterium*	2.57	4.50	1.78	3.14	1.18
*Lactobacillus*	7.99^b^	11.59^ab^	17.39^a^	16.05^ab^	17.01^a^
*Megasphaera*	1.35^ab^	3.77^a^	1.16^ab^	1.61^ab^	0.65^b^
*Ruminococcus*	0.47	0.62	0.53	0.60	0.74
*Streptococcus*	0.72	0.11	0.32	0.11	0.22
**Proteobacteria**					
*Escherichia-Shigella*	1.82^b^	5.68^ab^	8.84^ab^	7.49^ab^	15.90^a^
**d 14 PI**					
**Bacteroidota**					
*Muribaculaceae*	3.30	2.61	2.56	1.16	3.20
*Prevotella*	7.10^abc^	5.30^c^	10.66^a^	12.40^a^	8.16^ab^
**Firmicutes**					
*Agathobacter*	1.55	2.07	1.06	2.39	2.65
*Blautia*	11.75	11.07	7.51	8.79	8.22
*Coprococcus*	2.27^a^	2.93^ab^	1.17^b^	1.55^ab^	2.66^ab^
*Dorea*	2.39^ab^	2.08^abc^	3.26^a^	1.58^bc^	1.37^c^
*Faecalibacterium*	3.74	4.19	3.72	4.77	4.65
*Lactobacillus*	10.89	15.79	11.75	15.42	15.26
*Megasphaera*	1.67	3.53	3.46	2.39	2.02
*Ruminococcus*	1.00	1.76	1.53	1.83	1.79
*Streptococcus*	9.77^a^	3.88^a^	2.89^a^	5.21^a^	0.73^b^
**Proteobacteria**					
*Escherichia-Shigella*	0.10	0.24	0.03	0.15	0.02
**d 21 PI**					
**Bacteroidota**					
*Muribaculaceae*	2.98	3.03	4.19	3.19	5.28
*Prevotella*	9.86^a^	13.25^a^	8.59^ab^	12.37^a^	5.55^b^
**Firmicutes**					
*Agathobacter*	1.29	1.50	1.10	1.12	0.46
*Blautia*	6.90^ab^	5.08^b^	9.15^a^	6.38^ab^	5.20^b^
*Coprococcus*	2.41	2.25	1.36	1.29	1.58
*Dorea*	1.73^ab^	1.22^ab^	2.31^a^	1.71^ab^	0.82^b^
*Faecalibacterium*	2.66	2.37	2.31	3.25	1.72
*Lactobacillus*	9.11^ab^	7.33^ab^	12.38^a^	8.75^ab^	6.20^b^
*Megasphaera*	1.50^bc^	2.39^ab^	5.08^a^	4.73^a^	0.28^c^
*Ruminococcus*	1.48	1.98	1.49	2.24	1.61
*Streptococcus*	6.91^a^	5.96^ab^	2.41^bc^	4.66^ab^	0.18^c^
**Proteobacteria**					
*Escherichia-Shigella*	0.05	0.05	0.01	0.03	0.02

^a − c^Means without a common superscript are different (*P* < 0.05). Each mean represents 9–10 observations.

### Ileal digesta microbiota

**Figure 6:**
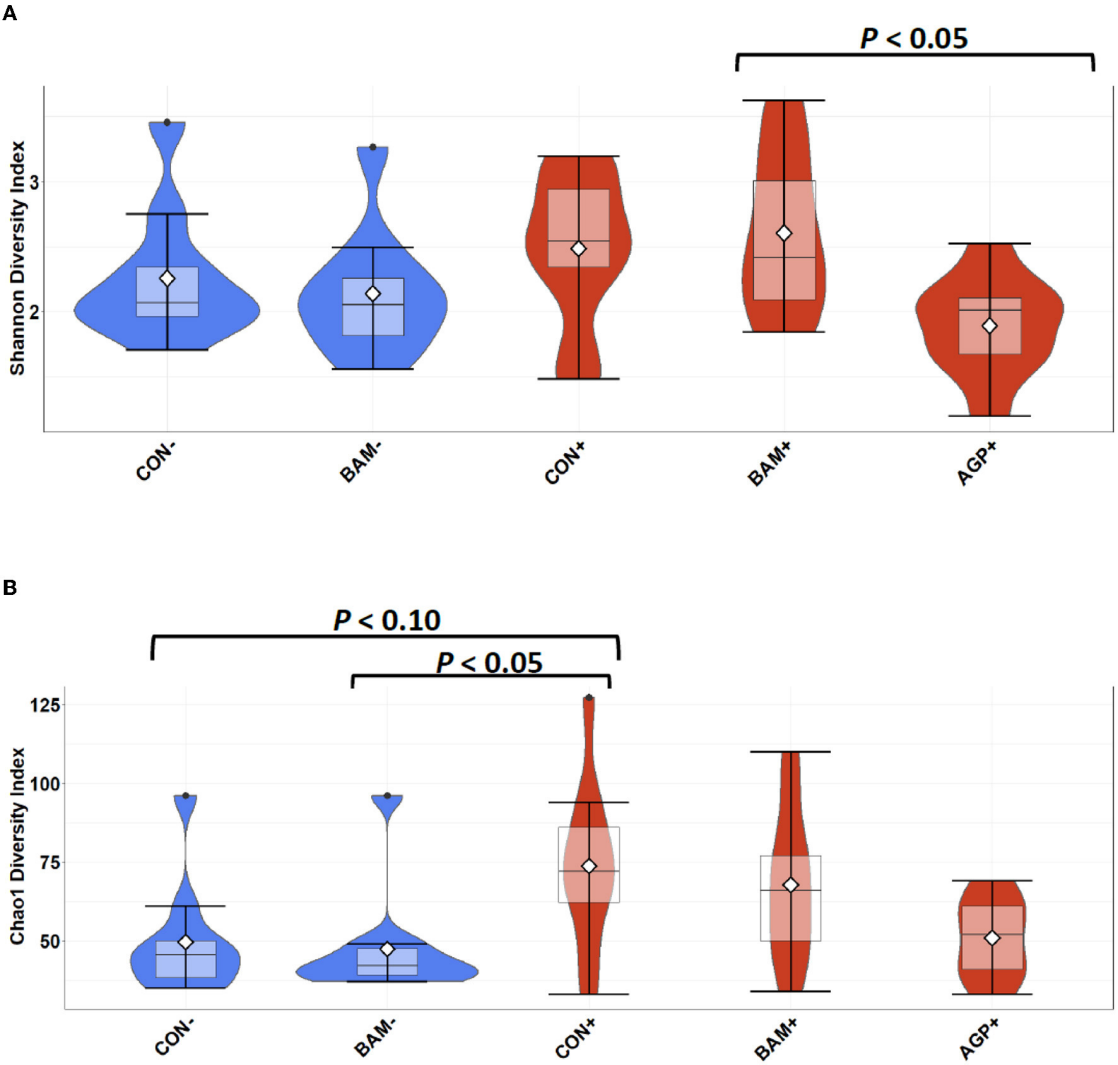
Alpha diversity as indicated by Shannon **(A)** and Chao1 **(B)** indices in ileal digesta collected from weaned pigs fed with a control (CON) diet or diets supplemented with *Bacillus amyloliquefaciens* (BAM) or antibiotics (AGP) 21 days after enterotoxigenic *E. coli* (ETEC) inoculation. Violin plots are colored by ETEC infected or not. Data are expressed as mean (diamond) ± SEM.

**Figure 7:**
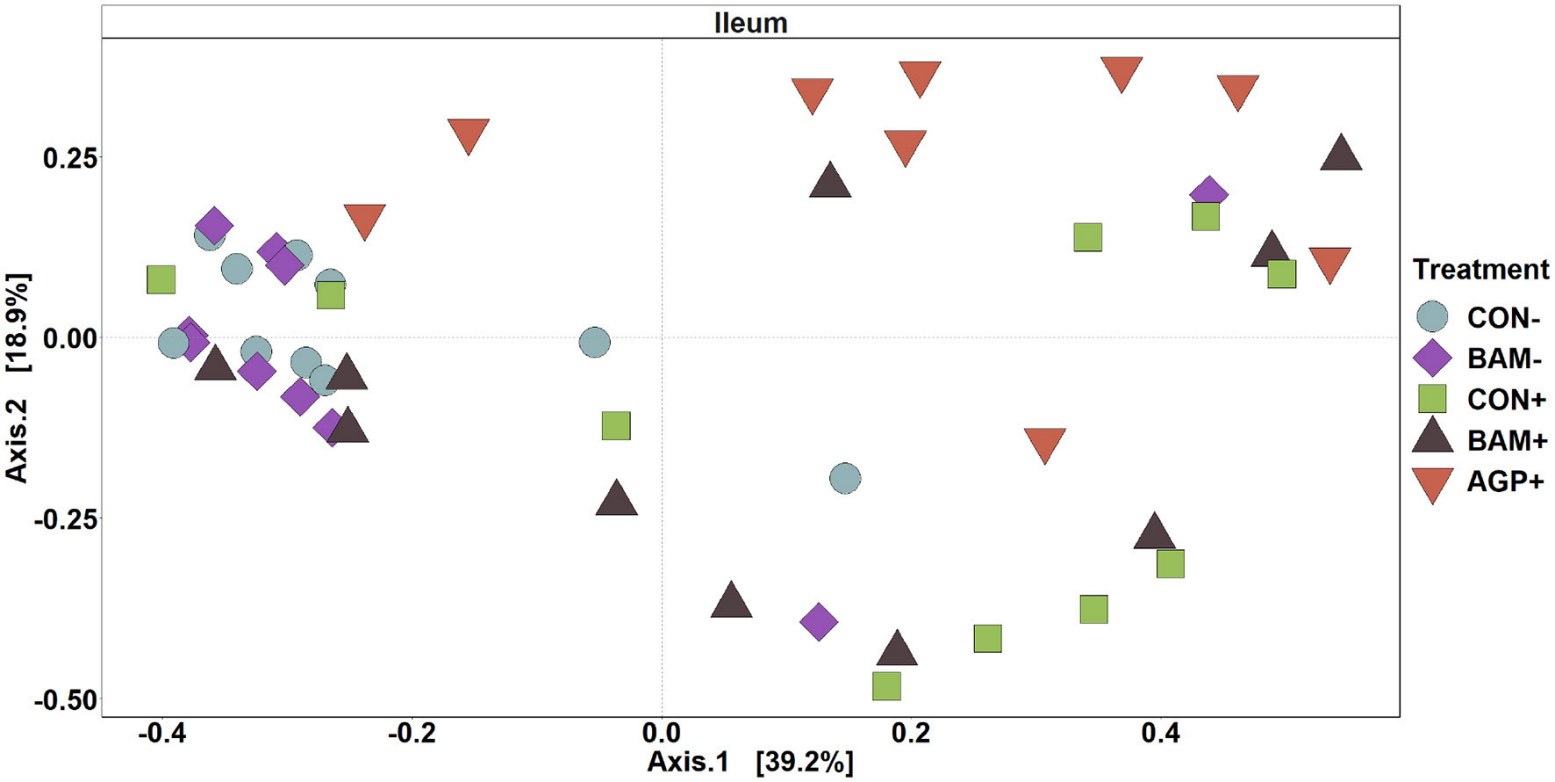
Principal coordinate analysis (PCoA) based on Bray-Curtis distance for beta diversity of ileal digesta of weaned pigs fed with a control (CON) diet or diets supplemented with *Bacillus amyliloquefaciens* (BAM) and antibiotics (AGP). Different symbols and shapes represent treatments. Each treatment has 9–10 observations.

In ileal digesta, BAM+ had a greater (*P* < 0.05) Shannon index than AGP+ (Figure 6A). Pigs in CON+ had a greater (*P* < 0.05) Chao1 diversity and a tendency (*P* < 0.10) to be greater than pigs in BAM- and CON-, respectively, in terms of comparing Chao1 diversity (Figure 6B). In beta diversity, CON- and BAM- clusters were overlapping each other and separated from ETEC-infected groups, while the ETEC-infected groups had ileal digesta samples that were more dispersed from each other (Figure 7). Clusters for BAM+ and CON+ were overlapping each other, and the AGP+ cluster was partially isolated from other treatment clusters.

The top four abundant phyla in ileal digesta collected on day 21 PI were Firmicutes, Proteobacteria, Actinomycetota, and Bacteroidota, from most to least abundant (Table 6). Pigs in CON+ had a greater (*P* < 0.05) relative abundance of Bacteroidota and Proteobacteria but a lower (*P* < 0.05) relative abundance of Firmicutes than pigs in CON-. At the family level, pigs in CON+ had a greater (*P* < 0.05) relative abundance of *Atopobiaceae, Clostridiaceae*, and *Pasteurellaceae* but a lower (*P* < 0.05) relative abundance of *Lactobacillaceae* than pigs in CON-. No difference was observed (*P* > 0.05) in ileal digesta microbiota between CON and BAM regardless of the ETEC challenge. Under the ETEC challenge, AGP enhanced (*P* < 0.05) the relative abundance of Firmicutes and *Clostridiaceae* but reduced (*P* < 0.05) the relative abundance of Bacteroidota and *Atopobiaceae* in ileal digesta compared with CON+. Compared with AGP+, BAM+ increased (*P* < 0.05) the relative abundance of Actinomycetota, Bacteroidota, *Atopobiaceae, Bifidobacteriaceae*, and *Prevotellaceae* but reduced (*P* < 0.05) the relative abundance of Firmicutes and *Clostridiaceae* in ileal digesta.

**Table 6:** Relative abundance (%) of Actinomycetota, Bacteroidota, Firmicutes, and Proteobacteria and their top families in ileal digesta of weaned pigs a fed control (CON) diet or diets supplemented with *Bacillus amyloliquefaciens* (BAM) or antibiotics (AGP).

	**Ileal digesta**				
	**Sham**		***Escherichia coli***		
	**CON-**	**BAM-**	**CON**+	**BAM**+	**AGP**+
Actinomycetota	3.38^ab^	3.85^ab^	4.62^ab^	9.84^a^	1.39^b^
*Atopobiaceae*	0.04^bc^	0.17^ab^	0.25^a^	2.11^a^	0.00^c^
*Bifidobacteriaceae*	3.28^ab^	3.58^ab^	4.02^ab^	7.50^a^	1.30^b^
Bacteroidota	0.02^b^	0.33^ab^	0.12^a^	1.80^a^	0.01^b^
*Prevotellaceae*	0.02^ab^	0.31^ab^	0.11^ab^	1.78^a^	0.01^b^
Firmicutes	94.52^a^	93.51^a^	76.48^b^	76.63^b^	92.68^a^
*Clostridiaceae*	2.14^cd^	3.71^d^	16.49^b^	16.4^bc^	49.23^a^
*Erysipelotrichaceae*	3.03	6.99	4.21	4.98	0.56
*Lactobacillaceae*	70.95^a^	69.31^a^	26.23^b^	31.51^b^	30.80^b^
*Peptostreptococcaceae*	6.73	3.58	4.60	6.76	1.81
*Streptococcaceae*	8.43	3.43	19.28	4.89	9.22
Proteobacteria	1.52^b^	2.07^b^	17.81^a^	10.93^ab^	4.35^ab^
*Enterobacteriaceae*	0.38	0.28	0.07	0.70	0.04
*Pasteurellaceae*	0.93^c^	1.78^bc^	17.58^a^	10.20^a^	4.00^ab^

^a−*d*^Means without a common superscript are different (*P* < 0.05). Each mean represents 9–10 observations.

Within the eight most abundant genera, one was under Actinomycetota, six were under Firmicutes, and one was under Proteobacteria (Table 7). ETEC infection increased (*P* < 0.05) the relative abundance of *Clostridium sensu stricto* 1 and *Actinobacillus* but reduced (*P* < 0.05) the relative abundance of *Lactobacillus* when comparing pigs in CON+ vs. CON-. AGP+ had a greater (*P* < 0.05) relative abundance of *Clostridium sensu stricto* 1 and less (*P* < 0.05) relative abundance in *Megasphaera* than CON+. In addition, the relative abundance of *Megasphaera* and *Bifidobacterium* was greater (*P* < 0.05) in BAM+ than in AGP+, while AGP+ had a greater (*P* < 0.05) relative abundance of *Clostridium sensu stricto* 1 than BAM+.

**Table 7:** Relative abundance (%) of most abundant genera from Firmicutes and Proteobacteria in ileal digesta of weaned pigs fed a control (CON) diet or diets supplemented with *Bacillus amyloliquefaciens* (BAM) or antibiotics (AGP).

	**Ileal digesta**				
	**Sham**		***Escherichia coli***		
	**CON-**	**BAM-**	**CON**+	**BAM**+	**AGP**+
**Actinomycetota**					
*Bifidobacterium*	3.26^ab^	3.42^ab^	4.02^ab^	7.45^a^	1.30^b^
**Firmicutes**					
*Clostridium sensu stricto* 1	2.07^c^	3.69^c^	16.46^b^	16.03^b^	49.06^a^
*Lactobacillus*	70.92^a^	69.31^a^	26.22^b^	31.51^b^	30.80^b^
*Megasphaera*	2.14^a^	3.99^a^	1.90^a^	5.18^a^	0.30^b^
*Streptococcus*	8.43	3.43	19.28	4.89	9.22
*Terrisporobacter*	4.71	2.63	2.23	3.93	1.80
*Turicibacter*	3.02	6.97	4.14	4.90	0.53
**Proteobacteria**					
*Actinobacillus*	0.90^c^	1.76^bc^	17.28^a^	10.00^a^	3.99^ab^

^a−*c*^Means without a common superscript are different (*P* < 0.05). Each mean represents 9–10 observations.

## Discussion

Antibiotics have been shown to induce prophages in the fecal samples of pigs over time, imposing a risk of developing antibiotic-resistant pathogens that could spread to humans (Allen et al., 2011). Complete eradication of antibiotic use at the post-weaning stage is desirable but currently not feasible; thus, developing alternative practices to treat post-weaning diarrhea and enhance the feed efficiency of weaned pigs is necessary (Angulo et al., 2005). Direct-fed microbials are looked upon to alleviate the intestinal damage caused by ETEC and to reduce the mortality rate of weaned pigs (Buntyn et al., 2016). Although *B. amyloliquefaciens* supplementations have been tested on broilers, limited studies have investigated the effects of *B. amyloliquefaciens* in weaned pigs. The present study observed that *B. amyloliquefaciens* supplementation tended to enhance growth performance and reduce systemic inflammation in weaned pigs challenged with ETEC F18. In addition, carbadox supplementation in the present study improved feed efficiency and alleviated diarrhea in ETEC-challenged weaned pigs. The gut microbiota were influenced differently between carbadox and *B. amyloliquefaciens*.

Consistent with our previous research, the increased frequency of diarrhea in pigs confirmed that the ETEC F18 strain inoculated into the pigs has successfully induced pathogenicity in the present study (Kim et al., 2019; He et al., 2020b). ETEC-infected pigs had reduced feed intake and weight gain and experienced severe diarrhea for approximately 6 days after the first ETEC inoculation, which falls under the average number of days when weaning pigs show diarrheal symptoms (Cox et al., 2012). No difference was observed in the growth performance of weaned pigs between the control and *B. amyloliquefaciens* in the sham group. Supplementation of *B. amyloliquefaciens* tended to enhance body weight, average daily gain, and feed intake of ETEC-infected pigs compared with controls. In consistency with previous research, supplementation of mixed strains of *B. amyloliquefaciens* enhanced feed efficiency of ETEC-infected pigs, while supplementation of a single strain of *B. amyloliquefaciens* reduced feed conversion ratio in broilers under necrotic enteritis challenge (Jerzsele et al., 2012; Becker et al., 2020). As expected, the supplementation of carbadox in the current study reduced the frequency of diarrhea and enhanced the growth rate of ETEC-challenged pigs. This observation was consistent with the results of Hung et al. (2020) and was supported by our fecal culture results, in which pigs fed with carbadox had fewer β-hemolytic coliforms in feces right after ETEC inoculation.

A complete blood count is crucial for evaluating the systemic severity of inflammation induced by bacterial infections, including ETEC. An increase in the number of white blood cells is commonly used to indicate the presence of systemic inflammation (Gordon-Smith, 2013). Our previous research also reported that the ETEC F18 challenge significantly increased the number of white blood cells, neutrophils, lymphocytes, and monocytes in weaned pigs at different time points (Liu et al., 2013; He et al., 2020a). In the present study, we also observed that ETEC inoculation increased total white blood cell counts and lymphocytes within 7 days post-inoculation. Reduced counts of lymphocytes and white blood cells in pigs supplemented with *B. amyloliquefaciens* or carbadox on days 14 and 21 PI suggest that both supplements may alleviate systemic inflammation caused by ETEC. These findings were also analogous to a study where *B. amyloliquefaciens* supplementation decreased white blood cell counts when broilers were challenged with lipopolysaccharides (Li et al., 2015). Without the ETEC challenge, *B. amyloliquefaciens* supplementation did not impact blood counts compared with negative controls. Similar results were also observed by Tang et al. (2018), in which no difference was observed in white and red blood cell counts and lymphocyte percentages when healthy laying hens were supplemented with *B. amyloliquefaciens*. The results from the current and previous studies suggest that *B. amyloliquefaciens* may have limited impacts on systemic immunity in animals when they are healthy.

The gut microbiota play an important role in reducing intestinal inflammation to promote a mutual relationship with the host (Lawley and Walker, 2013). Watery diarrhea induced by ETEC infection can disturb the gut microbiota, leading to difficulty suppressing inflammation (Bin et al., 2018). The alpha diversity in the present study showed an increase in microbial diversity and richness in fecal samples of ETEC-challenged pigs compared to sham pigs. This observation was contradicted with the findings by Pollock et al. (2018), in which fecal microbial diversity decreased over time when pigs were challenged with ETEC. The decreased microbial diversity observed by Pollock et al. (2018) may have occurred due to pigs being inoculated with ETEC at five different time points throughout the experiment, whereas pigs in the present study were inoculated with ETEC for 3 consecutive days after a 7-day adaptation period. However, the alpha diversity results in the present study agree with another study by Pollock et al. (2019), in which microbial diversity and richness in feces were not affected by the ETEC challenge. The increase in microbial diversity and richness in fecal samples over time and the overlapping samples in beta diversity between days 14 and 21 PI can be an indicator of maturity and stability in microbial diversity in pigs over time (Chen et al., 2017).

Firmicutes and Bacteroidota were the most abundant phyla in fecal microbiota throughout the experiment, which was expected in weaned piglets (Pajarillo et al., 2014; Yue et al., 2020; Luise et al., 2021). As expected, ETEC infection altered the microbial composition in the feces of pigs. The relative abundance of *Escherichia–Shigella* peaked 7 days after the first ETEC inoculation and then decreased on day 14 PI. This observation was supported by a gradual decrease in the diarrhea severity of ETEC-infected pigs in the present study, and it agrees with the results of Kim et al. (2022) that pigs underwent recovery from ETEC infection around 11 days after inoculation. Carbadox has been shown to decrease the relative abundance of taxa that are noted to be highly abundant in the fecal microbiota of pigs, which includes *Lachnospiraceae, Blautia*, and *Lactobacillus*. Carbadox is known to be a bactericidal primarily active against gram-positive bacteria; however, the mechanism behind this is yet to be known (Constable et al., 2017). In agreement with Lourenco et al. (2021), carbadox supplementation in the current study significantly decreased the relative abundance of *Agathobacter* on day 7 PI, *Dorea* and *Streptococcus* on day 14 PI, and *Blautia* and *Dorea* on day 21 PI, which are all gram-positive bacteria. Although some taxa, including *Enterobacteriaceae* and *Clostridiaceae*, are still abundant after carbadox exposure, the changes in gut bacterial biomass are unknown due to the limits of 16S rRNA sequencing. Moreover, the decrease in these taxa was observed on days 7 and 21 PI, indicating that carbadox may induce short- and long-term shifts in the fecal microbiota of weaned pigs by reducing microbial diversity (Holman et al., 2017). Supplementation of *B. amyloliquefaciens* had limited effects on fecal microbiota in pigs in the sham group. However, supplementation of *B. amyloliquefaciens* moderately affected fecal microbiota composition compared with control or carbadox under the ETEC challenge. On day 14 PI, pigs fed with *B. amyloliquefaciens* had less abundant *Dorea* than pigs in the positive control but had more abundant *Streptococcus* than pigs fed with carbadox. Supplementation of *B. amyloliquefaciens* also increased the relative abundance of *Prevotella, Megasphaera*, and *Streptococcus* compared with pigs fed with carbadox on day 21 PI.

Microbiota changes in the ileal digesta on day 21 PI were expected when pigs were challenged with ETEC or fed different diets. Unlike the proximate site of the digestive system, which has more bacterial barrier including stomach acid and bile salts, the ileum provides an optimal environment for ETEC proliferation in pigs (Gonzales et al., 2013; Roussel et al., 2020). Similar to the alpha diversity results in fecal microbiota, ETEC infection tended to increase microbial diversity and richness in the ileum. These findings were also concurrent with the findings by Pollock et al. (2019). The ETEC challenge in the current study decreased the relative abundance of Firmicutes but increased the relative abundance of Bacteroidota and Proteobacteria phyla in the ileum. The ratio of Firmicutes to Bacteroidota is widely accepted to play an important role in maintaining normal intestinal homeostasis, and a decrease in the Firmicutes:Bacteroidota ratio is usually observed in inflammatory bowel disease (Shen et al., 2018). However, this ratio can also be affected by an increase in other phyla during dysbiosis, such as the change in Proteobacteria. Growing evidence suggests that Proteobacteria is the most variable phylum, which contributes to microbial perturbation and may lead to increased disease risks (Morgan et al., 2012; Shin et al., 2015). The changes in ileal phyla are also explained by the changes in microbiota composition at family and genera levels. ETEC infection reduced the relative abundance of *Lactobacillus* (26.22% in positive control vs. 70.92% in negative control) but increased the relative abundance of *Clostridium sensu stricto* 1 and *Actinobacillus* in ileal digesta. Various *Lactobacillus* species have shown beneficial impacts on overall intestinal ecology; thus, the species are commonly being investigated as probiotic candidates in humans and pigs (de Vries et al., 2006; Suo et al., 2012; Sayan et al., 2018). It was also reported that commensal *Lactobacillus* can activate the host immunity to promote the overall health of mice (Holman et al., 2017; Nakamoto et al., 2017; Qi et al., 2019). *Actinobacillus* are gram-negative bacteria with most of the species characterized as commensals, but some species are considered pathogens in animal disease and the abundance was reported to increase in human disease as well (Rycroft and Garside, 2000; Denoth et al., 2021). *Clostridium sensu stricto* 1 is characterized as an opportunistic pathogen, which was reported to induce intestinal inflammation and reduce short-chain fatty acid production in pigs and poultry (Yang et al., 2019; Li et al., 2020; Hu et al., 2021). Thus, a reduction in *Lactobacillus* abundance and an increase in *Actinobacillus* and *Clostridium sensu stricto* 1 in the ileal digesta of ETEC-infected pigs confirm that ETEC infection remarkably disturbs the intestinal microbiota community of weaned pigs by potentially competing for colonization sites or nutrients with favorable bacteria. The present results also suggest that ETEC can cause a long-term perturbation in ileal microbiota throughout the weaning phase of pigs. Perturbation in the ileal microbiota may lead to unfavorable consequences in pig health, including immunosuppression and disrupted integrity in intestinal structure (Xia et al., 2022). In addition, more differences were observed in ileal microbiota than in fecal microbiota, which is likely due to the ileum being the major site of ETEC colonization (Martín-Rodríguez et al., 2022).

Under the ETEC challenge, pigs supplemented with *B. amyloliquefaciens* had an increased microbial diversity and relative abundance of Actinomycetota, particularly *Bifidobacterium* in the ileal digesta than pigs fed with carbadox. Actinomycetota plays an important role in maintaining gut homeostasis, especially their genus *Bifidobacterium* is also commonly investigated as potential probiotics, as they support the host immune system by stimulating the release of immunoglobulins in the intestinal mucosa (Holman et al., 2017; Binda et al., 2018; Sun et al., 2020). The presence of *B. amyloliquefaciens* may aid the growth of other microbes in the gut to outperform ETEC colonization (Dubreuil et al., 2016). Moreover, *B. amyloliquefaciens* supplementation modified the ileal microbiota differently compared with carbadox. Compared with *B. amyloliquefaciens*, carbadox further increased the abundance of *Clostridium sensu stricto* 1 and reduced the abundance of *Prevotellaceae* in ileal microbiota, which was also observed in a pig study with antibiotic growth promoter tylosin and a mouse study with enrofloxacin (Kim et al., 2016; Sun et al., 2019). The increased abundance of Firmicutes under carbadox supplementation may build on existing evidence of Firmicutes developing antimicrobial resistance genes due to consistent exposure to antibiotics (Anthony et al., 2022). Current results also suggest that carbadox supplementation may have long-term impacts on the ileal microbiota of weaned pigs, which may not be able to reestablish similarly to that of healthy weaned pigs (Yue et al., 2020). Our findings highlight taxa influenced by ETEC infection and/or dietary supplements; however, future studies should consider evaluating the functional genomes from the gut microbiota and assess the relationship between growth performance and immunity of the host to their microbiota.

## Conclusion

As the use of antibiotic growth promoters becomes less desirable globally, alternative practices are imperative for enhancing growth and reducing post-weaning diarrhea in weaned pigs. The present study ultimately observed that supplementing *B. amyloliquefaciens* tended to increase feed intake and weight gain but had limited impacts on the diarrhea of weaned pigs infected with ETEC. However, pigs fed with *B. amyloliquefaciens* had relatively milder systemic inflammation than controls under disease-challenging conditions. In addition, pigs supplemented with *B. amyloliquefaciens* had a relatively higher abundance of *Bifidobacterium* but lower *Clostridium sensu stricto* 1 than carbadox when pigs were challenged with ETEC. The modulatory effects of *B. amyloliquefaciens* on immunity and ileal microbiota in pigs warrant further investigation. Taken altogether, supplementation of *B. amyloliquefaciens* solely may not provide growth enhancement and acute diarrheal alleviation of weaned pigs as similar to the addition of carbadox. Although various *Bacillus* spp. have shown the potential for promoting animal health and performance, findings in the present study suggest that there are differences between *Bacillus* spp. and their effects on different animal species. Nevertheless, manipulating the gut microbiota to overall improve pig health and eradicate ETEC pathogenicity is currently a captivating interest in the swine industry. To further assess the importance of gut microbiota to alleviate post-weaning diarrhea, future studies are suggested to employ metagenomics and to investigate correlations among gut microbiota, immunity, and growth performance of pigs undergoing diarrhea in a larger scale study.

## Data availability statement

The datasets presented in this study can be found in online repositories. The names of the repository/repositories and accession number(s) can be found below: https://www.ncbi.nlm.nih.gov/, PRJNA887417.

## Ethics statement

The protocol for this experiment was reviewed and approved by the Institutional Animal Care and Use Committee (IACUC# 20809) at the University of California, Davis (UC Davis).

## Author contributions

YL designed and supervised the entire experiment. CJ conducted the animal experiment and performed sample analysis and data analysis. CJ, MK, and YL contributed to the result interpretation. BW and XL provided assistance for animal experiments. CJ and YL wrote the manuscript. BW, MK, and XL revised the manuscript. The final version was approved by all authors.
